# SparkBLAST: scalable BLAST processing using in-memory operations

**DOI:** 10.1186/s12859-017-1723-8

**Published:** 2017-06-27

**Authors:** Marcelo Rodrigo de Castro, Catherine dos Santos Tostes, Alberto M. R. Dávila, Hermes Senger, Fabricio A. B. da Silva

**Affiliations:** 1 Computer Science Department, Federal University of São Carlos, Rod. Washington Luís, Km 235, São Carlos, 21040-900 Brazil; 2 LBCS-IOC, Oswaldo Cruz Foundation, Av Brasil 4365, Rio de Janeiro, 21040-900 Brazil; 3 PROCC, Oswaldo Cruz Foundation, Av. Brasil 4365, Rio de Janeiro, 21040-900 Brazil

**Keywords:** Cloud computing, Comparative genomics, Scalability, Spark

## Abstract

**Background:**

The demand for processing ever increasing amounts of genomic data has raised new challenges for the implementation of highly scalable and efficient computational systems. In this paper we propose SparkBLAST, a parallelization of a sequence alignment application (BLAST) that employs cloud computing for the provisioning of computational resources and Apache Spark as the coordination framework. As a proof of concept, some radionuclide-resistant bacterial genomes were selected for similarity analysis.

**Results:**

Experiments in Google and Microsoft Azure clouds demonstrated that SparkBLAST outperforms an equivalent system implemented on Hadoop in terms of speedup and execution times.

**Conclusions:**

The superior performance of SparkBLAST is mainly due to the in-memory operations available through the Spark framework, consequently reducing the number of local I/O operations required for distributed BLAST processing.

**Electronic supplementary material:**

The online version of this article (doi:10.1186/s12859-017-1723-8) contains supplementary material, which is available to authorized users.

## Background

Sequence alignment algorithms are a key component of many bioinformatics applications. The NCBI BLAST [1, 2] is a widely used tool that implements algorithms for sequence comparison. These algorithms are the basis for many other types of BLAST searches such as BLASTX, TBLASTN, and BLASTP [3]. The demand for processing large amounts of genomic data that gushes from NGS devices has grown faster than the rate which industry can increase the power of computers (known as Moore’s Law). This fact has raised new challenges for the implementation of scalable and efficient computational systems. In this scenario, MapReduce (and its Hadoop implementation) emerged as a paramount framework that supports design patterns which represent general reusable solutions to commonly occurring problems across a variety of problem domains including analysis and assembly of biological sequences [4]. MapReduce has delivered outstanding performance and scalability for a myriad of applications running over hundreds to thousands of processing nodes [5]. On the other hand, over the last decade, cloud computing has emerged as a powerful platform for the agile and dynamic provisioning of computational resources for computational and data intensive problems.

Several tools have been proposed, which combine Hadoop and cloud technologies. Regarding NGS we can cite Crossbow [6] and for sequence analysis: Biodoop [7] and CloudBLAST [8]. Further tools based on Hadoop and related technologies are surveyed in [4].

Despite of its popularity, MapReduce requires algorithms to be adapted according to such design patterns [9]. Although this adaptation may result in efficient implementations for many applications, this is not necessarily true for many other algorithms, which limits the applicability of MapReduce. Moreover, because MapReduce is designed to handle extremely large data sets, its implementation frameworks (e.g. Hadoop and the Amazon’s Elastic MapReduce service) constrains the program’s ability to process smaller data.

More recently, Apache Spark has emerged as a promising and more flexible framework for the implementation of highly scalable parallel applications [10, 11]. Spark does not oblige programmers to write their algorithms in terms of the map and reduce parallelism pattern. Spark implements in-memory operations, based on the Resilient Distribution Datasets (RDDs) abstraction [11]. RDD is a collection of objects partitioned across nodes in the Spark cluster so that all partitions can be computed in parallel. We may think of RDDs as a collection of data objects which are transformed into new RDDs as the computation evolves. Spark maintains lists of dependencies among RDDs which are called “lineage”. It means RDDs can be recomputed in case of lost data (e.g. in the event of failure or simply when some data has been previously discarded from memory).

In this paper we propose SparkBLAST, which uses the support of Apache Spark to parallelize and manage the execution of BLAST either on dedicated clusters or cloud environments. Spark’s *pipe* operator is used to invoke BLAST as an external library on partitioned data of a query. All the input data (the query file and the database) and output data of a query are treated as Spark’s RDDs. SparkBLAST was evaluated on both Google and Microsoft Azure Clouds, for several configurations and dataset sizes. Experimental results show that SparkBLAST improves scalability when compared to CloudBLAST in all scenarios presented in this paper.

## Implementation

A design goal is to offer a tool which can be easily operated by users of the unmodified BLAST. Thus, SparkBLAST implements a driver application written in Scala, which receives user commands and orchestrates the whole application execution, including data distribution, tasks execution, and the gathering of results in a transparent way for the user.

**Fig. 1:**
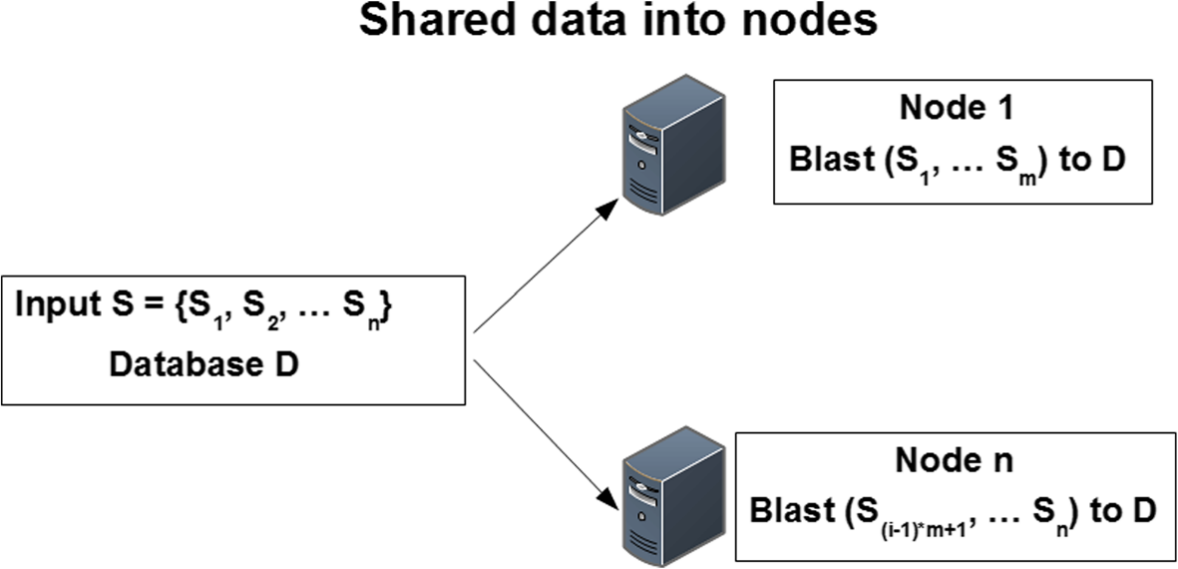
Data distribution among *n* nodes: the target database (*D*) is copied on every computing node; the query file (*S*) is evenly partitioned into data splits (*S*_1_,…,*S*_*n*_) which are distributed over the nodes. Each split (*S*_*i*_) can be replicated on more than one node for fault tolerance

Two input files must be provided for a typical operation: (*i*) the target database of bacterial genomic sequences, which will be referred to as *target database* from now on, for short; and (*ii*) the *query file*, which contains a set of query genomic sequences that will be compared to the target’s database sequences for matching. As depicted in Fig. 1, SparkBLAST replicates the entire target database on every computing node. The query file is evenly partitioned into data *splits* which are distributed over the nodes for the execution. Thus, each computing node has a local deployment of the BLAST application, and it receives a copy of the entire target database and a set of fragments of the query file (splits).

Note that it is possible to apply different techniques for task and data partitioning. Each data split (i.e., fragment of the query file) can be replicated by the distributed file system (DFS) on a number of nodes, for fault tolerance purposes. Spark’s scheduler then partitions the whole computation into tasks, which are assigned to computing nodes based on data locality using delay scheduling [12]. For the execution of each task, the target database and one fragment of the query file are loaded in memory (as RDDs). The target database (RDD) can be reused by other local tasks that execute in the same machine, thus reducing disk access [11].

SparkBLAST uses Spark Pipe to invoke the local installation of the NCBI BLAST2 on each node, and execute multiple parallel and distributed tasks in the cluster.

Spark can execute on top of different resource managers, including Standalone, YARN, and Mesos [13]. We chose YARN because it can be uniformly used by Spark and Hadoop. It is important to avoid the influence of resource scheduling in the performance tests presented in this paper. In fact, YARN was originally developed for Hadoop version 2. With YARN, resources (e.g., cpu, memory) can be allocated and provisioned as *containers* for tasks execution on a distributed computing environment. It plays better the role of managing the cluster configuration, and dynamically shares available resources, providing support for fault tolerance, inter-, and intra-node parallelism. Other applications which have been written or ported to run on top of YARN include Apache HAMA, Apache Giraph, Open MPI, and HBASE^1^.

**Fig. 2:**
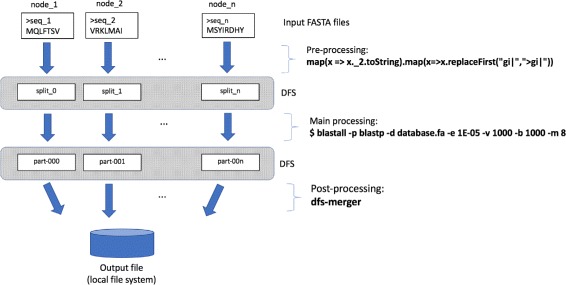
The workflow implemented by SparkBLAST: during each of the three stages, parallel tasks (represented as *vertical arrows*) are executed in the computing nodes. Pre-processing produce the splits of the query file and copy them to the DFS. The main processing execute local instances of BLAST on local data. Finally, the post processing merges output fragments into a unique output file

Data processing in SparkBLAST can be divided into three main stages (as depicted in Fig. 2): pre-processing, main processing and post-processing. Such stages are described in the following subsections.

### Execution environment

In order to evaluate the performance and the benefits of SparkBLAST, we present two experiments. The first experiment was executed in the Google Cloud, and the second experiment executed in the Microsoft Azure platform. Both experiments executed with 1, 2, 4, 8, 16, 32, and 64 virtual machines as computing nodes for scalability measurement. For the sake of comparison, each experiment was executed on SparkBLAST and on CloudBLAST. The later is a Hadoop based tool designed to support high scalability on clusters and cloud environments. For the experiments, we used Spark 1.6.1 to execute SparkBLAST on both cloud environments. To execute CloudBLAST, we used Hadoop 2.4.1 on the Google Cloud, and Hadoop 2.5.2 on Azure Cloud. In any case, we configures YARN as the resource scheduler, since our experiments focus on performance. Further details on the experimental setup will be provided in the results section.

### Input data generation

This work was originally inspired and applied in a radionuclides resistance study. Genome sequences of several radiation-resistant microorganisms can be used for comparative genomics to infer the similarities and differences among those species. Homology inference is important to identify genes shared by different species and, as a consequence, species-specific genes can be inferred. Two experiments are considered in this work. The input data for Experiment 1 was composed of 11 bacterial genome protein sequences, 10 of these are radiation-resistant (*Kineococcus radiotolerans* - Accession Number NC_009660.1, *Desulfovibrio desulfuricans* - NC_011883.1, *Desulfovibrio vulgaris* - NC_002937.3, *Rhodobacter sphaeroides* - NC_009429.1, *Escherichia coli* - NC_000913.3, *Deinococcus radiodurans* - NC_001263.1, *Desulfovibrio fructosivorans* - NZ_AECZ01000069.1, *Shewanella oneidensis* - NC_004349.1, *Geobacter sulfurreducens* - NC_002939.5, *Deinococcs geothermalis* - NC_008010.2, *Geobacter metallireducens* - NC_007517.1) for Reciprocal-Best-Hit (RBH) processing.

For Experiment 2, the input query is composed of 10 radiation-resistant bacteria. (i.e., all species listed above but *E. coli*). This similarity-based experiment consisted on the search of potential protein homologs of 10 radiation-resistant genomes in 2 marine metagenomics datasets.

Each input dataset was concatenated into a single multifasta input file named query1.fa (Experiment 1) and query2.fa (Experiment 2). The files query1.fa and query2.fa had 91,108 and 86,968 sequences and a total size of 36.7 MB and 35 MB, respectively. Two target metagenomic datasets obtained from MG-RAST database^2^ were used in Experiment 2: (i) Sargaso Sea (Bermuda), coordinates: 32.17,-64.5, 11 GB, 61255,260 proteins (Ber.fasta) and (ii) João Fernandinho (Buzios, Brazil), coordinates: -22.738705, -41874604, 805 MB, 4795,626 proteins (Buz.fasta):



### Pre-processing

In this stage, implemented by SparkBLAST, the query file is evenly partitioned into splits which are written to the DFS. The splits are then distributed among the computing nodes by the DFS, according to some replication policy for fault tolerance. Each split containing a set of (e.g., thousands of) genome sequences can be processed by a different task. Thus, the query file should be partitioned to enable parallelism. Since the input file can be potentially large, the partitioning operation can be also parallelized as illustrated in the following commands:



### Main processing

This stage starts after all the input data (i.e., the target database and query file splits) are properly transferred to each processing node. Tasks are then scheduled to execute on each node according to data locality. The amount of tasks executed concurrently on each computing node depends on the number of processing cores available. As soon as a computing core completes the execution of a task, it will be assigned another task. This process repeats until the available cores execute all tasks of the job.

During this stage, each individual task uses Spark pipe to invoke a local execution of BLASTP as illustrated by the following command line:



Note that the query input file to be processed has been omitted because it varies for each task.

In order to measure the scalability and *speedup* of SparkBLAST we carried out experiments on both the Google Cloud and Microsoft Azure, increasing the platform size from 1 to 64 computing nodes. For the sake of comparison, the same genome searches have been executed with both SparkBLAST and CloudBLAST for each platform size. Every experiment was repeated six times and and the average execution time was considered in results.

For the sake of reproducibility, both experiments with SparkBLAST and CloudBLAST were executed with the following configuration parameters:





Therefore, each node will act as a *mapper*, producing outputs similar to the unmodified BLAST.

### Post-processing

During the previous stage each individual task produces a small output file. During the post-processing stage, SparkBLAST merges all these small files into a single final output file. For instance, experiment 1 produces a final output file of 610 MB. All output data is written to the DFS, i.e., the Google Cloud Storage or Microsoft Azure’s Blob storage service.

Added-value to SparkBLAST, similarity results were obtained by (i) performing a Reciprocal Best Hit analysis [14, 15] among pairs of species, or orthology inference (Experiment 1) and (ii) searching for potential radiation-resistant homologous proteins in 2 marine metagenome datasets (Experiment 2), as described in the following section.

## Results

In order to assess the performance and benefits of SparkBLAST, we carried out experiments on two cloud platforms: Google Cloud and Microsoft Azure. The same executions were carried out on both SparkBLAST and CloudBLAST.

### Results for experiment 1 - executed on the Google Cloud

In Experiment 1, BLASTP was used to execute queries on a 36 MB database composed of 88,355 sequences from 11 bacterial genomes, in order to identify genes shared by different species. Ten bacteria described in literature as being resistant to ionizing radiation [16] and one species susceptible to radiation were obtained from Refseq database. The same dataset is provided as query and target database, so that an all-to-all bacteria comparison is executed, producing a 610 MB output. BLASTP results were processed to identify RBH among pairs of species.

Experiment 1 was executed on a platform with up to 64 computing nodes plus one master node. Each node is a virtual machine configured as *n1-standard-2* instance (2 vCPUs, 7.5 GB memory, CPU Intel Ivy Bridge). The virtual machines were allocated from 13 different availability zones in the Google Cloud: Asia East (3 zones), Europe West (3 zones), US Central (4 zones) e US East (3 zones). For this scalability test, both SparkBLAST and CloudBLAST were executed on platforms with 1, 2, 4, 8, 16, 32, and 64 nodes. The experiment was repeated six times for each platform size. Thus, Experiment 1 encompasses 2×7×6=84 executions in total, which demanded more than 350 h (wall clock) to execute. As an estimate on the amount of the required computational resources, this experiment consumed 2.420 vCPU-hours to execute on the Google Cloud.

**Fig. 3:**
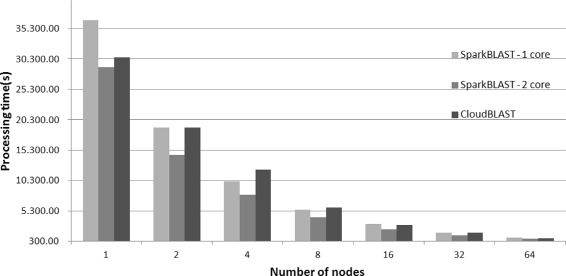
Total execution time for CloudBLAST *vs.* SparkBLAST running on the Google Cloud. Values represent the average of six executions for each experiment

**Fig. 4:**
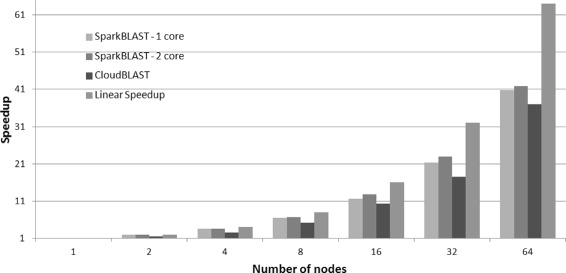
Speedup for 1 to 64 nodes in the Google Cloud. SparkBlast was executed on virtual machines with one and two cores. CloudBlast was executed on nodes with two cores

The average execution times are presented in Fig. 3. SparkBLAST achieved a maximum speedup (which is the ratio between execution time of the one node baseline over the run time for the parallel execution) of 41.78, reducing the execution time from 28,983 s in a single node, to 693 s in 64 nodes. In the same scenario, CloudBLAST achieved speedup of 37, reducing the execution time from 30,547 to 825 s on 64 nodes. For this set of executions, both SparkBLAST and CloudBLAST used 2 vCPUs per node for tasks execution. The speedup is presented in Fig. 4. As shown, SparkBLAST presents better scalability than CloudBLAST.

The average execution times and standard deviations are presented in Table 1. Table 2 presents the execution times for SparkBLAST when only one vCPU (core) of each node is used for processing. Table 3 presents the total execution times for SparkBLAST when both cores of each node are used for processing.

**Table 1:** Execution times for CloudBLAST - Google Cloud

# nodes	1	2	4	8	16	32	64
Exec. time 1	29,921.40	19,018.00	11,324.00	6,204.00	2,866.00	1,680.00	794.00
Exec. time 2	30,256.23	18,550.25	13,799.23	5,779.21	2,959.65	1,828.23	900.00
Exec. time 3	31.016.85	19,221.81	12,580.32	5,700.52	3,004.52	1,597.00	815.21
Exec. time 4	31.350.25	19,102.68	10,489.53	5,850.02	2,961.23	1,806.25	842.30
Exec. time 5	30.726.89	18,981.32	12,721.23	5,780.34	2,990.81	1,780.32	799.21
Exec. time 6	30.012.14	19,118.72	11,820.85	5,900.64	3,008.15	1,753.23	802.98
Mean	30,547.29	18,998.80	12,122.53	5,869.12	2,965.06	1,740.84	825.62
Std. Dev.	576.25	235.28	1.164.02	177.70	52.79	87.20	40.32
Std.Dev./Mean	1.89%	1.24%	9.60%	3.03%	1.78%	5.01%	4.88%

**Table 2:** Execution times - SparkBLAST 1 core - Google Cloud

# nodes	1	2	4	8	16	32	64
Exec. time 1	36,106.86	18,845.23	10,189.11	5,556.22	3,129.20	1,716.10	905.21
Exec. time 2	36,510.12	19,120.32	10,199.85	5,540.15	3,115.12	1,730.58	899.84
Exec. time 3	36,720.86	18,952.15	10,170.23	5,560.88	3,140.01	1,790.96	894.76
Exec. time 4	38,120.25	18,998.06	10,200.01	5,543.62	3,120.58	1,694.69	900.42
Exec. time 5	36,230.56	19,112.23	10,178.76	5,552.10	3,122.15	1,701.55	897.65
Exec. time 6	36,452.53	18,880.11	10,183.61	5,565.11	3,127.58	1,710.68	890.25
Mean	36,690.20	18,984.68	10,186.93	5,553.01	3,125.77	1,724.09	898.02
Std.Dev	733.00	115.14	11.83	9.73	8.62	35.01	5.14
Std.Dev/Mean	2.00%	0.61%	0.12%	0.18%	0.28%	2.03%	0.57%

**Table 3:** Execution times - SparkBLAST 2 cores - Google Cloud

# nodes	1	2	4	8	16	32	64
Exec. time 1	28,915.52	14,500.86	7,935.45	4,287.85	2,249.94	1,260.12	695.23
Exec. time 2	29,002.21	14,520.23	7,945.10	4,290.12	2,230.26	1,259.28	690.04
Exec. time 3	29,001.89	14,515.35	7,950.01	4,283.56	2,255.04	1,260.10	701.50
Exec. time 4	28,989.52	14,557.51	7,942.20	4,282.21	2,242.63	1,259.52	710.11
Exec. time 5	28,990.32	14,580.01	7,940.80	4,310.12	2,249.26	1,259.82	680.80
Exec. time 6	29,001.15	14,520.23	7,950.12	4,295.56	2,251.08	1,262.15	682.10
Mean	28,983.44	14,532.37	7,943.95	4,291.57	2,246.37	1,260.17	693.30
Std.Dev	33.78	29.93	5.68	10.27	8.85	1.03	11.37
Std.Dev/Mean	0.12%	0.21%	0.07%	0.24%	0.39%	0.08%	1.64%

Table 4 consolidates results from previous tables and presents mean execution times along with speedup and parallel efficiency figures for the CloudBLAST and SparkBLAST (1 and 2 cores) systems.

**Table 4:** Mean execution times, speedups and parallel efficiency (Experiment 1 - query.fasta - 36 MB) - SparkBLAST vs CloudBLAST - Google Cloud

# nodes	1	2	4	8	16	32	64
SparkBLAST							
1 core							
Exec. time	36,690.20	18,984.68	10,186.93	5,553.01	3,125.77	1,724.09	898.02
Speedup	1	1.93	3.60	6.61	11.74	21.28	40.86
Efficiency	1	0.97	0.90	0.83	0.73	0.67	0.64
SparkBLAST							
2 cores							
Exec. time	28,983.44	14,532.37	7,943.95	4,291.57	2,246.37	1,260.17	693.30
Speedup	1.00	1.99	3.65	6.75	12.90	23.00	41,81
Efficiency	1.00	1.00	0.91	0.84	0.81	0.72	0.65
CloudBLAST							
Exec. time	30,547.29	18,998.80	12,122.53	5,869.12	2,965.06	1,740.84	825.62
Speedup	1.00	1.61	2.52	5.20	10.30	17.55	37.00
Efficiency	1.00	0.80	0.63	0.65	0.64	0.55	0.58

**Fig. 5:**
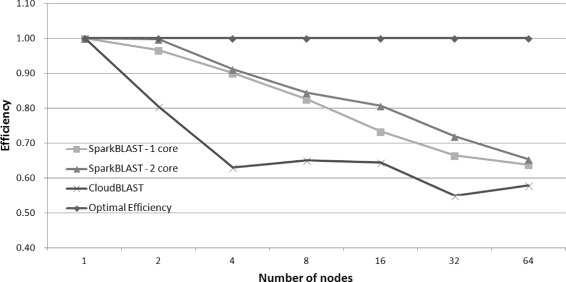
Efficiency for CloudBLAST x SparkBLAST running on Google Cloud

Figure 3 compares total execution times of CloudBLAST and SparkBLAST (one and two cores configurations), for platforms composed of 1 up to 64 computing nodes. Execution times presented in correspond to the average for six executions. Parallel efficiency is presented in Fig. 5.

### Results for experiment 2 - executed on the Microsoft Azure

Experiment 2 was executed on a total of 66 nodes allocated on the Microsoft Azure Platform, being all nodes from the same location (East-North US). Two A4 instances (8 cores and 14 GB memory) were configured as master nodes, and 64 A3 (4 cores and 7 GB memory) instances were configured as computing nodes. Both SparkBLAST and CloudBLAST executed queries on two datasets (Buz.fasta, and Ber.fasta), varying the number of cores allocated as 1 (BLAST sequential execution), 4, 12, 28, 60, 124 and 252. Every execution was repeated 6 times for CloudBLAST and six times for SparkBLAST. Thus, Experiment 2 encompasses 2×2×7×6=168 executions in total, which demanded more than 8,118 h (wall clock) to execute. An estimate on the amount of computational resources, this experiment consumed more than 139,595 vCPU-hours to execute on the Azure Cloud.

**Fig. 6:**
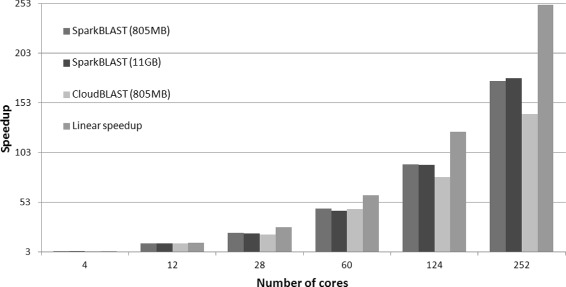
Speedup - Microsoft Azure

**Fig. 7:**
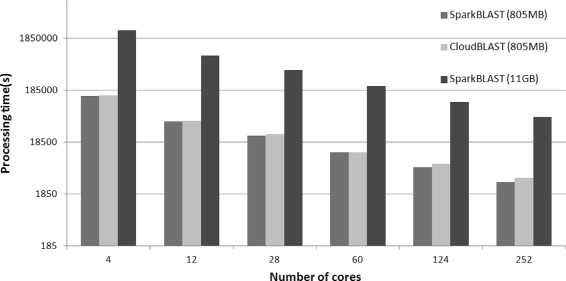
Total execution time for CloudBLAST x SparkBLAST on Microsoft Azure

**Fig. 8:**
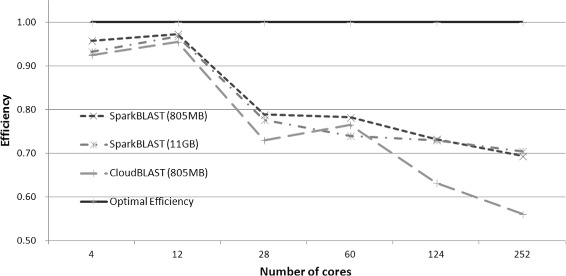
Efficiency - CloudBLAST x SparkBLAST - Microsoft Azure

For the Microsoft Azure platform, SparkBLAST outperforms CloudBlast on all scenarios. Both datasets (Buz.fasta and Ber.fasta) were processed, and results are presented in Fig. 6 (speedup), Fig. 7 (total execution time), Fig. 8 (Efficiency), Table 5 (Buz.fasta), and Table 6 (Ber.fasta). It is worth noting that the largest dataset (Ber.fasta - 11 GB) was larger than the available memory in the computing nodes. For this reason, CloudBLAST could not process the Ber.fasta dataset, while SparkBLAST does not have this limitation. It is also worth mentioning that larger speedups were achieved on Microsoft Azure when compared to the Google Cloud. This can be partially explained by the fact that all computing nodes allocated on the Microsoft Azure are placed in the same location, while computing nodes on Google Cloud were distributed among 4 different locations.

**Table 5:** Mean execution times, speedups and parallel efficiency (Experiment 2 - Buz.fasta - 805 MB) - SparkBLAST vs CloudBLAST - Microsoft Azure

# cores	4	12	28	60	124	252
SparkBLAST	143,228.95	47,031.62	24,850.51	11,692.45	6,041.64	3,138.64
Speedup	3.83	11.67	22.09	46.95	90.86	174.89
Efficiency	0.96	0.97	0.79	0.78	0.73	0.69
CloudBLAST	148,512.95	47,950.05	26,858.71	11,951.11	6,993.52	3,879.06
Speedup	3.7	11.45	20.44	45.93	78.49	141.51
Efficiency	0.92	0.95	0.73	0.77	0.63	0.56

**Table 6:** Mean execution times, speedups and parallel efficiency (Experiment 2 - Ber.fasta - 11 GB) - SparkBLAST vs CloudBLAST - Microsoft Azure

# cores	4	12	28	60	124	252
SparkBLAST	2,678,902.06	859,687.13	458,759.75	224,869.12	110,222.98	56,200.21
Speedup	3.73	11.61	21.76	44.4	90.57	177.64
Efficiency	0.93	0.97	0.78	0.74	0.73	0.7
CloudBLAST	-	-	-	-	-	-
Speedup	-	-	-	-	-	-
Efficiency	-	-	-	-	-	-

### Similarity-based inferences

In order to obtain added-value from the SparkBLAST similarity results on the cloud, the output from SparkBLAST processing of Experiment 1 was used to infer orthology relationships with the RBH approach. In Table 7, numbers represent (RBH) orthologs found between 2 species. Numbers in bold represent (RBH) paralogs found in the same species. The higher number of RBH shared by two species was 264 between *Desulfovibrio vulgaris* and *Desulfovibrio desulfuricans*, and the lower was 15 between *Desulfovibrio fructosivorans* and *Deinococcus radiodurans*. Among the same species, the higher number of RBH was 572 in *Rhodobacter sphaeroides* and the lower 34 in *Deinococcus geothermalis*. Regarding experiment 2: 1.27% (778,349/61255,260) of the Bermuda metagenomics proteins and 1.4% (68,748/4795,626) of the Búzios metagenomic proteins represent hits or potential homologs to the 10 radiation-resistant bacteria.

**Table 7:** Numbers of RBH found using data from SparkBLAST cloud processing

*Kineococcus*	*Desulfovibrio*	*Desulfovibrio*	*Rhodobacter*	*Escherichia*	*Deinococcus*	*Desulfovibrio*	*Shewanella*	*Geobacter*	*Deinococcus*	*Geobacter*	Name	Accession	Number of
*radiotolerans*	*desulfuricans*	*vulgaris*	*sphaeroides*	*coli*	*radiodurans*	*fructosivorans*	*oneidensis*	*sulfurreducens*	*geothermalis*	*metallireducens*		Number	proteins
**224**	43	25	63	35	53	22	21	18	52	20	*Kineococcus*	NC_009660.1	4,632
											*radiotolerans*		
	**380**	264	121	79	38	163	71	88	27	60	*Desulfovibrio*	NC_011883.1	10,443
											*desulfuricans*		
		**362**	62	46	17	98	53	47	24	37	*Desulfovibrio*	NC_002937.3	12,349
											*vulgaris*		
			**572**	114	46	46	98	77	50	44	*Rhodobacter*	NC_009429.1	20,954
											*sphaeroides*		
				**98**	24	26	155	34	28	26	*Escherichia*	NC_000913.3	4,140
											*coli*		
					**122**	15	21	27	102	17	*Deinococcus*	NC_001263.1	7,671
											*radiodurans*		
						**84**	20	40	17	38	*Desulfovibrio*	NZ_AECZ01000069.1	4,028
											*fructosivorans*		
							**90**	36	21	32	*Shewanella*	NC_004349.1	8,271
											*oneidensis*		
								**146**	20	120	*Geobacter*	NC_002939.5	9,340
											*sulfurreducens*		
									**34**	16	*Deinococcs*	NC_008010.2	2,935
											*geothermalis*		
										**50**	*Geobacter*	NC_007517.1	3,592
											*metallireducens*		

Numbers in bold represent (RBH) 354 paralogs found in the same species

## Discussion

In this paper we investigate the parallelization of sequence alignment algorithms through an approach that employs cloud computing for the dynamic provisioning of large amounts of computational resources and Apache Spark as the coordination framework for the parallelization of the application. SparkBLAST, a scalable parallelization of sequence alignment algorithms is presented and assessed. Apache Spark’s *pipe* operator and its main abstraction RDD (*resilient distribution dataset*) are used to perform scalable protein alignment searches by invoking BLASTP as an external application library. Experiments on the Google Cloud and Microsoft Azure have demonstrated that the Spark-based system outperforms a state-of-the-art system implemented on Hadoop in terms of speedup and execution times. It is worth noting that SparkBLAST can outperform CloudBlast even when just one of the vCPUs available per node is used by SparkBLAST, as demostrated by results obtained on the Google Cloud. In the experiments presented in this paper, the Hadoop-based system always used all vCPUs available per node.

From Table 4 it is possible to verify that both Speedup and Parallel Efficiency are better for SparkBLAST when compared to CloudBLAST for experiments executed on both the Google Cloud and Microsoft Azure. This is true for both scenarios of SparkBLAST on the Google Cloud (1 and 2 cores per node). It is worth noting that even when total execution time for CloudBLAST is smaller than the 1-core SparkBLAST configuration, Speedup and Parallel Efficiency is always worse for CloudBLAST. When SparkBLAST allocates two cores per node (as CloudBLAST does) execution times are always smaller when compared to CloudBLAST.

For the Microsoft Azure platform, all measures (processing time, efficiency and speedup) are better on SparkBLAST when compared to the corresponding execution of CloudBLAST for the Buz.fasta (805 MB) dataset. It is worth noting that the speedup difference in favor of SparkBLAST increases with the number of computing nodes, which highlights the improved scalability of SparkBLAST over CloudBLAST. As mentioned in the “Results” section, it was not possible to process the larger Ber.fasta (11 GB) dataset using CloudBLAST due to computing node’s main memory limitation. This constraint does not affect SparkBLAST, which can process datasets even when they are larger than the main memory available on computing nodes. In the case of Spark, every process invoked by a task (each core is associated to a task) can use RDD even when database do not fit in memory, due memory content reuse and the implementation of circular memory [17]. It is worth noting that RDDs are stored as deserialized Java objects in the JVM. If the RDD does not fit in memory, some partitions will not be cached and will be recomputed on the fly each time they are needed [10]. Indeed, one very important loophole of existing methods that we address in SparkBLAST is the capability of processing large files on the Cloud. As described in this paragraph, SparkBLAST can process much larger files when compared to CloudBLAST, due to a more efficient memory management.

The main reason behind the performance of SparkBLAST when compared to Hadoop-based systems are the in-memory operations and its related RDD abstraction. The reduced number of Disk IO operations by SparkBLAST results in a significant improvement on overall performance when compared to the Hadoop implementation.

It is clear that in-memory operations available in SparkBLAST plays a major role both in Speedup and Parallel Efficiency improvements and, as a consequence, also in the scalability of the system. Indeed, the main reason behind the fact that SparkBLAST, even when it allocates only half of nodes processing capacity, achieves performance figures that are superior of those of CloudBLAST is the reduced number of local I/O operations.

Another point to be highlighted is the scalability of SparkBLAST on a worldwide distributed platform such as Google Cloud. For the executions presented in this work, 64 nodes were deployed in 13 zones and it was achieved a speedup of 41.78 in this highly distributed platform. Once again, in-memory operations is a major factor related to this performance.

For applications where the Reduce stage is not a bottleneck, which is the case for SparkBLAST, it is demonstrated in the literature that Spark is much faster than Hadoop. In [18], those authors state that, for this class of application, MapReduce Hadoop is much slower than Spark in task initialization and is less efficient in memory management. Indeed, the supplementary document “Execution Measurements of SparkBLAST and CloudBLAST”, available in the online version of this paper, presents several measurements performed during SparkBLAST and CloudBLAST executions on the Microsoft Azure Cloud. These measurements show that task initialization in SparkBLAST is considerably faster than CloudBLAST. It is also shown that SparkBLAST is more efficient in memory management than CloudBLAST. The effect of SparkBLAST’s more efficient memory management can be observed in Additional file 1: Figures S5 and S6 of the supplementary information document. These figures show that Hadoop needs to use more memory than Spark, while Spark can maintain a larger cache and less swap to execute. Both factors - task initialization and memory management - are determinant for the improved scalability of SparkBLAST.

Furthermore, CloudBLAST makes use of Hadoop Streaming. In [19], authors shown that the Hadoop Streaming mechanism used in CloudBLAST can decrease application performance because it makes use of OS pipes to transfer input data to the applications’ (in this case BLAST) standard input and from BLAST standard output to disk storage. Data input to BLAST is done by the option: “-inputreader org.apache.hadoop.streaming. StreamPatternRecordReader”, which send lines from the input file to BLAST one-by-one, which further degrades performance.

Regarding extended scalability over larger platforms than the ones considered in this paper, it should be highlighted that two authors of this paper have proposed a formal scalability analysis of MapReduce applications [5]. In this analysis the authors prove that the most scalable MapReduce applications are reduceless applications, which is exactly the case of SparkBLAST. Indeed, Theorem 5.2 of [5] states that the increase of amount of computation necessary for a reduceless Scalable MapReduce Computation (SMC) application to maintain a given isoefficiency level is proportional to the number of processors (nodes). This is the most scalable configuration over all scenarios analyzed in [5]. Simulation results that goes up to 10000 nodes corroborate the limits stated in this and other theorems of [5].

Regarding Experiment 1 and RBH inference, we showed that our SparkBLAST results can be post-processed to infer shared genes, then generating added-value to the similarity analysis. That also means that RBH experiments using SparkBLAST are potentially scalable to many more genomes, and can be even used as part of other Blast-based homology inference software such as OrthoMCL [20]. Considering Experiment 2, results indicate that 1.27% of the Bermuda metagenomics proteins and 1.4% of the Búzios metagenomic proteins represent potential homologs to the 10 radiation-resistant bacteria, and as far as we know no related studies have been published to date. Those potential homologs will be further investigated in another study.

## Conclusion

In this paper we propose SparkBLAST, a parallelization of BLAST that employs cloud computing for the provisioning of computational resources and Apache Spark as the coordination framework. SparkBLAST outperforms CloudBLAST, a Hadoop-based implementation, in speedup, efficiency and scalability in a highly distributed cloud platform. The superior performance of SparkBLAST is mainly due to the in-memory operations available through the Spark framework, consequently reducing the number of local I/O operations required for distributed BLAST processing.

## Availability and requirements

-**Project name:** SparkBLAST;

-**Project home page:** https://github.com/sparkblastproject/v2

-**Operating system(s):** Debian 8.1 and Ubuntu Server 14.02

-**Programming language:** Scala, Shell and Java

-**Other requirements:**

-**Licence:** BSD 3-clause Clear License

## Endnotes

^1^ https://wiki.apache.org/hadoop/PoweredByYarn

^2^ http://metagenomics.anl.gov/

## Additional file

Additional file 1 Execution Measurements of SparkBLAST and CloudBLAST. In this supplementary document we present performance data collected during the execution of Experiment 2 on the Microsoft Azure Platform. **Figure S1.** CPU utilization for the SparkBLAST execution. **Figure S2.** CPU utilization for the CloudBLAST execution. **Figure S3.** CPU utilization for one worker node running SparkBLAST. **Figure S4.** CPU utilization for one worker node running CloudBLAST. **Figure S5.** Memory utilization for SparkBLAST. **Figure S6.** Memory utilization for CloudBLAST. **Figure S7.** Network traffic produced by SparkBLAST during its execution. **Figure S8.** Network traffic produced by CloudBLAST during its execution. (PDF 173 kb)

## Abbreviations

DFS: Distributed file system
NGS: Next generation sequencing
RDD: Resilient distribution datasets
RBH: Reciprocal best hits
SMC: Scalable MapReduce computation
vCPU: Virtual CPU
