# Supplementary material for: SparkBLAST: scalable BLAST processing using in-memory operations
Source: BMC Bioinformatics. 2017 Jun 27;18:318. doi: 10.1186/s12859-017-1723-8 (PMC5488373; doi:10.1186/s12859-017-1723-8)
Supplement: Additional file 1 — Execution Measurements of SparkBLAST and CloudBLAST. In this supplementary document we present performance data collected during the execution of Experiment 2 on the Microsoft Azure Platform. Figure S1. CPU utilization for the SparkBLAST execution. Figure S2. CPU utilization for the CloudBLAST execution. Figure S3. CPU utilization for one worker node running SparkBLAST. Figure S4. CPU utilization for one worker node running CloudBLAST. Figure S5. Memory utilization for SparkBLAST. Figure S6. Memory utilization for CloudBLAST. Figure S7. Network traffic produced by SparkBLAST during its execution. Figure S8. Network traffic produced by CloudBLAST during its execution. (PDF 173 kb) [file 12859_2017_1723_MOESM1_ESM.pdf]

Execution Measurements of SparkBLAST and CloudBLAST

Supplementary Information for SparkBLAST: Scalable BLAST processing using in-memory operations

Marcelo R. de Castro   Catherine dos Santos Tostes
Alberto M. R. Dávila   Hermes Senger   Fabrício A. B. Silva

June 2017

1 Supplementary Data on Experiments

In this supplementary document, we present performance data collected during the execution of Experiment 2 on the Microsoft Azure Platform. All nodes were placed in the same location (East-North US). The cluster is composed of two A4 instances (8 cores and 14GB memory) configured as master nodes and 64 A3 (4 cores and 7GB memory) instances configured as computing nodes. For the measurements presented in this text, both SparkBLAST and CloudBLAST executed queries on the Buz.fasta (805 MB) dataset. We used Galglia [Massie et al. 2004], a scalable distributed monitoring system, to collect the performance data that are presented in this supplementary document.

2 CPU utilization

The cluster average CPU utilization is presented in Figures S1 and S2. As illustrated, in both cases CPU utilization reaches near 97-98% after the initialization of the application. CPU utilization did not reach 100% because the master nodes are taken into account and they have low CPU utilization. Individual workers for both systems reach 100% CPU utiilization as depicted in Figure S3 and Figure S4.

As reported in our paper, the execution of CloudBLAST takes a sightly longer time than the SparkBLAST execution. There are some possible reasons for the extra overhead caused by Hadoop. First, Hadoop is much slower than Spark in task initialization [Shi et al. 2015]. Second, there is an overhead caused by the pipe and I/O operations implemented by Hadoop [Ding et al. 2011]. Hadoop uses Linux pipes to redirect *standard input* and *standard output* of BLAST to connect it to the Mapreduce environment. Pipes are inter process communication mechanisms that involve the creation of buffers between two communication processes. The producer process copies data into the buffer, while the consumer process collects and removes data items from the buffer. Both communicating processes have to synchronize. Lastly, the use of data type casting and unbuffered Java I/O operations, which write data to be accesed by external executables (e.g. BLAST) and read data from them, may also lead to performance overhead.

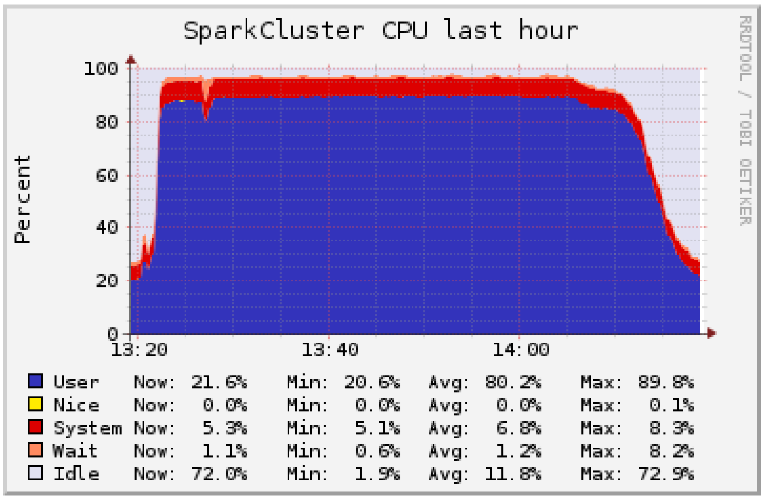

Figure S1: CPU utilization for the SparkBLAST execution.

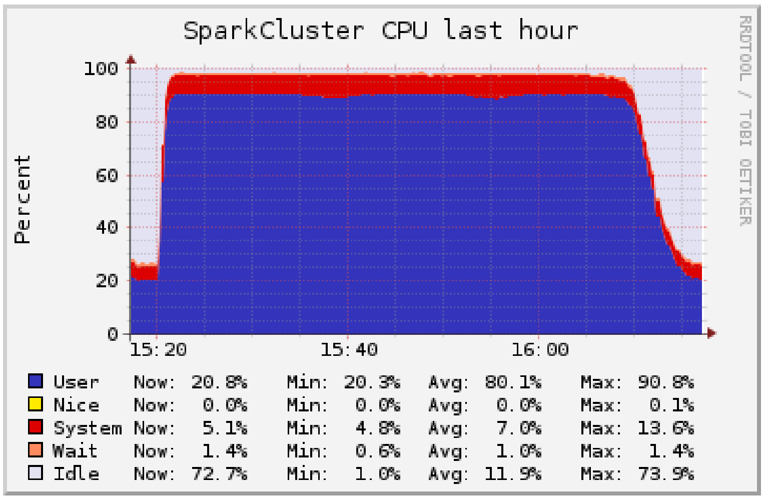

Figure S2: CPU utilization for the CloudBLAST execution.

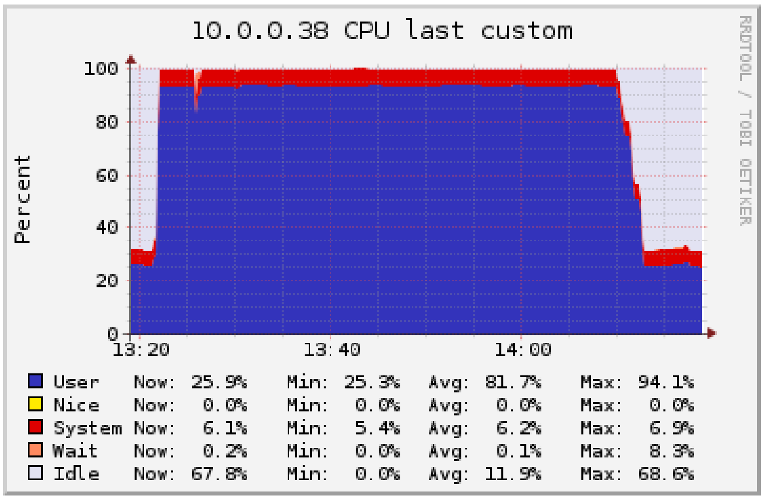

Figure S3: CPU utilization for one worker node running SparkBLAST.

3 Memory utilization

Another cause for superior performance of SparkBLAST is memory management. Spark implements the Resilient Distributed Datasets (RDDs), which implement in-memory data structures used to cache intermediate data across a set of nodes. The effect can be observed in Figures S5 and S6. Hadoop needs to use more memory than Spark, while Spark can maintain a larger cache and less swap to execute.

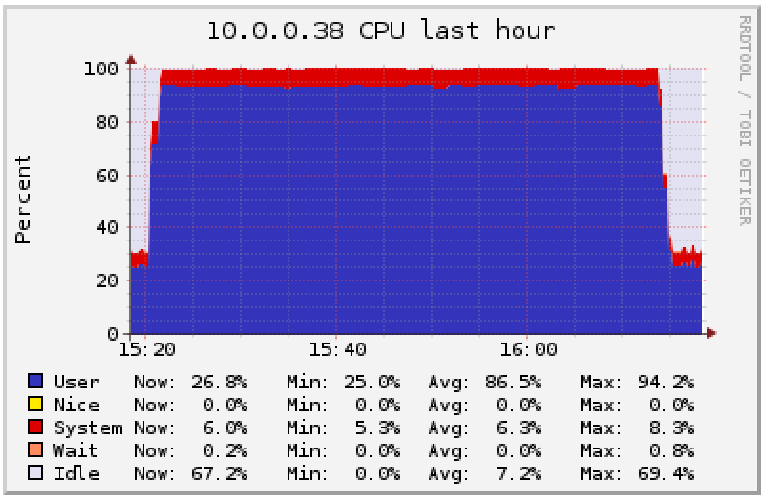

Figure S4: CPU utilization for one worker node running CloudBLAST.

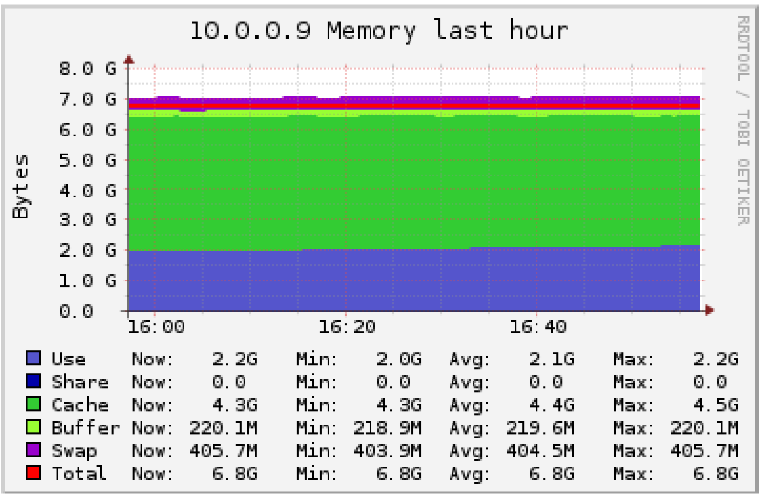

Figure S5: Memory utilization for SparkBLAST

4 Network traffic

The network traffic produced by SparkBLAST and CloudBLAST are presented in Figure S7 and Figure S8, respectively. The total amount of data transmitted over the network by SparkBLAST during its execution is 3.76 GB, while CloudBLAST's traffic reaches 8.92 GB. CloudBLAST experienced a traffic peak in the beginning of the execution because it transfers the data from the blob service to the local disk.

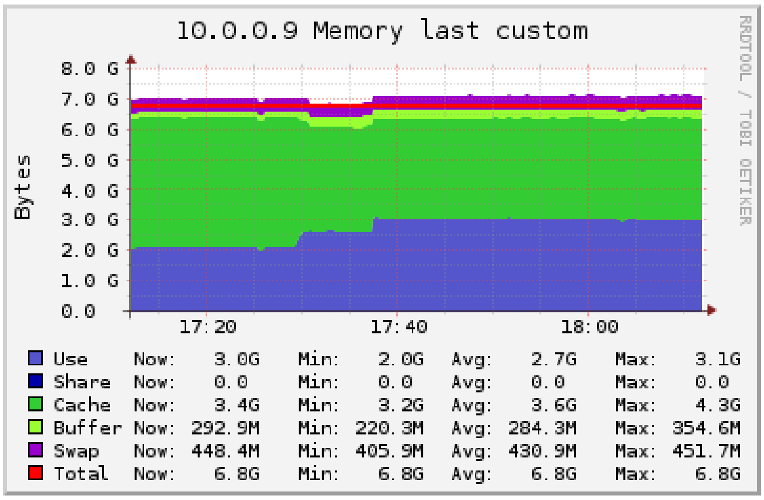

Figure S6: Memory utilization for CloudBLAST.

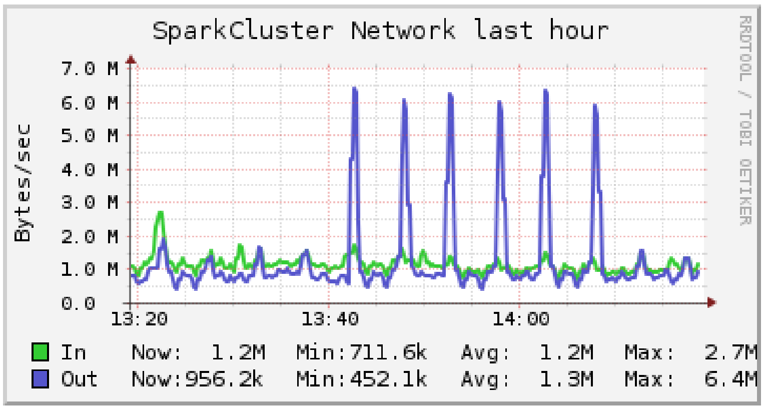

Figure S7: Network traffic produced by SparkBLAST during its execution.

5 Final Remarks

Although Hadoop and Spark are designed to support the execution of distributed applications on large clusters, their design and implementation lead to different performance. Compared to Hadoop, Spark has demonstrated better performance for the execution of BLAST applications on a cloud platform. Possible reasons for the superior performance of Spark come from its low overhead in task management, better memory utilization and less network traffic. Also, the underlying mechanisms used to implement Hadoop streaming also lead to extra overhead.

References

[Ding et al. 2011] Ding, M., Zheng, L., Lu, Y., Li, L., Guo, S., and Guo, M. (2011). More convenient more overhead: the performance evaluation of hadoop streaming. In *Proceedings of the 2011 ACM Symposium on Research in Applied Computation*, pages 307–313. ACM.

[Massie et al. 2004] Massie, M. L., Chun, B. N., and Culler, D. E. (2004). The ganglia distributed monitoring system: design, implementation, and experience. *Parallel Computing*, 30(7):817–840.

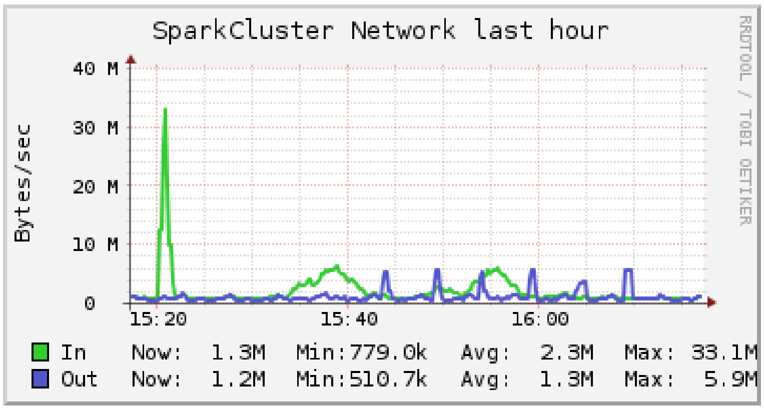

Figure S8: Network traffic produced by CloudBLAST during its execution.

[Shi et al. 2015] Shi, J., Qiu, Y., Minhas, U. F., Jiao, L., Wang, C., Reinwald, B., and Özcan, F. (2015). Clash of the titans: Mapreduce vs. spark for large scale data analytics. *Proc. VLDB Endow.*, 8(13):2110–2121.
